# Supplementary material for: Oxidation of two cysteines within yeast Hsp70 impairs proteostasis while directly triggering an Hsf1-dependent cytoprotective response
Source: J Biol Chem. 2022 Aug 27;298(10):102424. doi: 10.1016/j.jbc.2022.102424 (PMC9508553; doi:10.1016/j.jbc.2022.102424)
Supplement: Supplemental Figures S1–S5 [file mmc1.docx]

**Santiago and Morano**

**Supplementary Information**

**
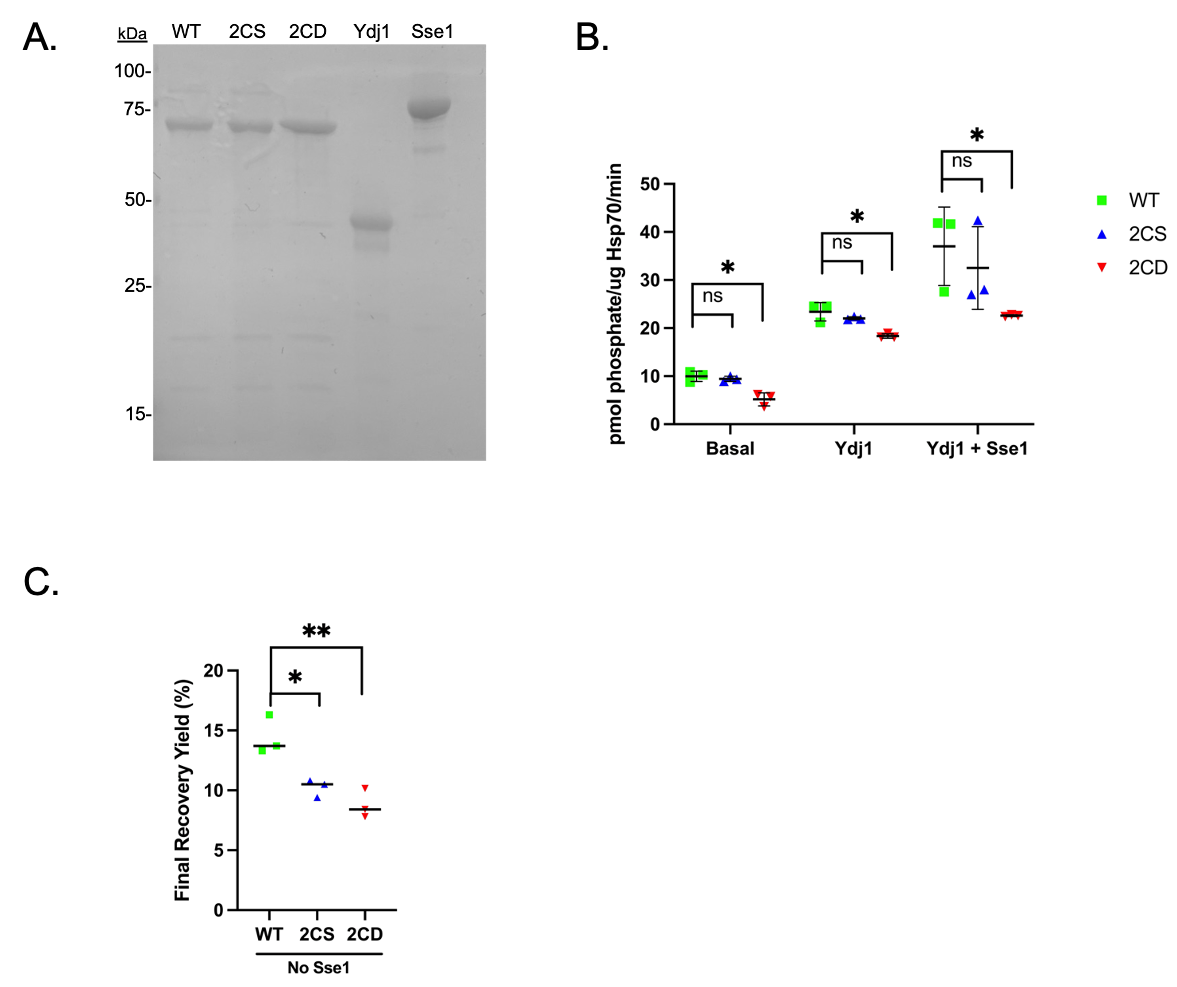
**

**Fig. S1. The oxidomimetic Ssa1-2CD mutant exhibits reduced basal and Ydj1-stimulated ATPase activity.** (A) Coomassie staining of approximately equivalent amounts of the indicated proteins used in the *in vitro* assays. (B) ATP hydrolysis by wild type and mutant Ssa1 proteins, basal and stimulated by 0.2 µM Ydj1. (C) Firefly luciferase (FFL) recovery over time in the presence of 0.1 uM of the indicated Ssa1 protein and the co-chaperone Ydj1 (0.2 uM), as described in detail in *Materials and Methods.* Bolded horizontal bars indicate mean, and error bars indicate SEM. *, p<0.05; ns, not significant.

**
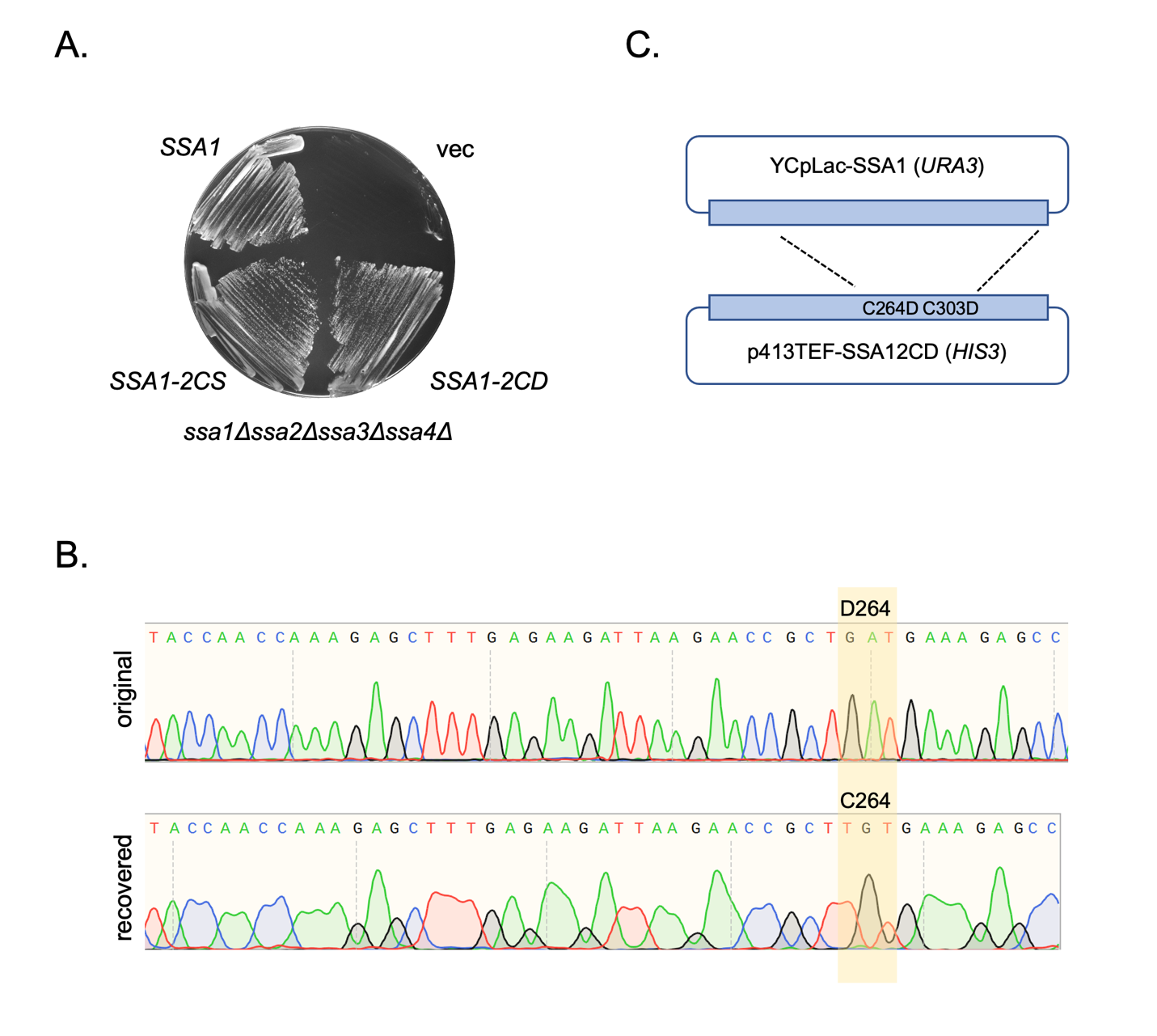
**

**Fig. S2.** **The oxidomimetic *ssa1-2CD* mutant is incapable of supporting viability as the sole cytosolic *SSA* gene.** (A) 48-hour 5-fluoroorotic acid (5-FOA) plate growth of the indicated *HIS3-*selective plasmid, in an *ssa1Δ ssa2Δ* *ssa3Δ ssa4Δ* deletion background demonstrating unexpected wild type growth for the *ssa1-2CD* mutant. (B) Sequencing analysis of the introduced *HIS3*-selective plasmid (original) and the recovered plasmid extracted from the colonies shown in (A), demonstrating reversion of the aspartic acid-encoding codon GAT to the original cysteine-encoding codon TGT. Only the region surrounding C264 is shown for simplicity. (C) Proposed recombination mechanism to explain the gene reversion event during the plasmid shuffle process.

**
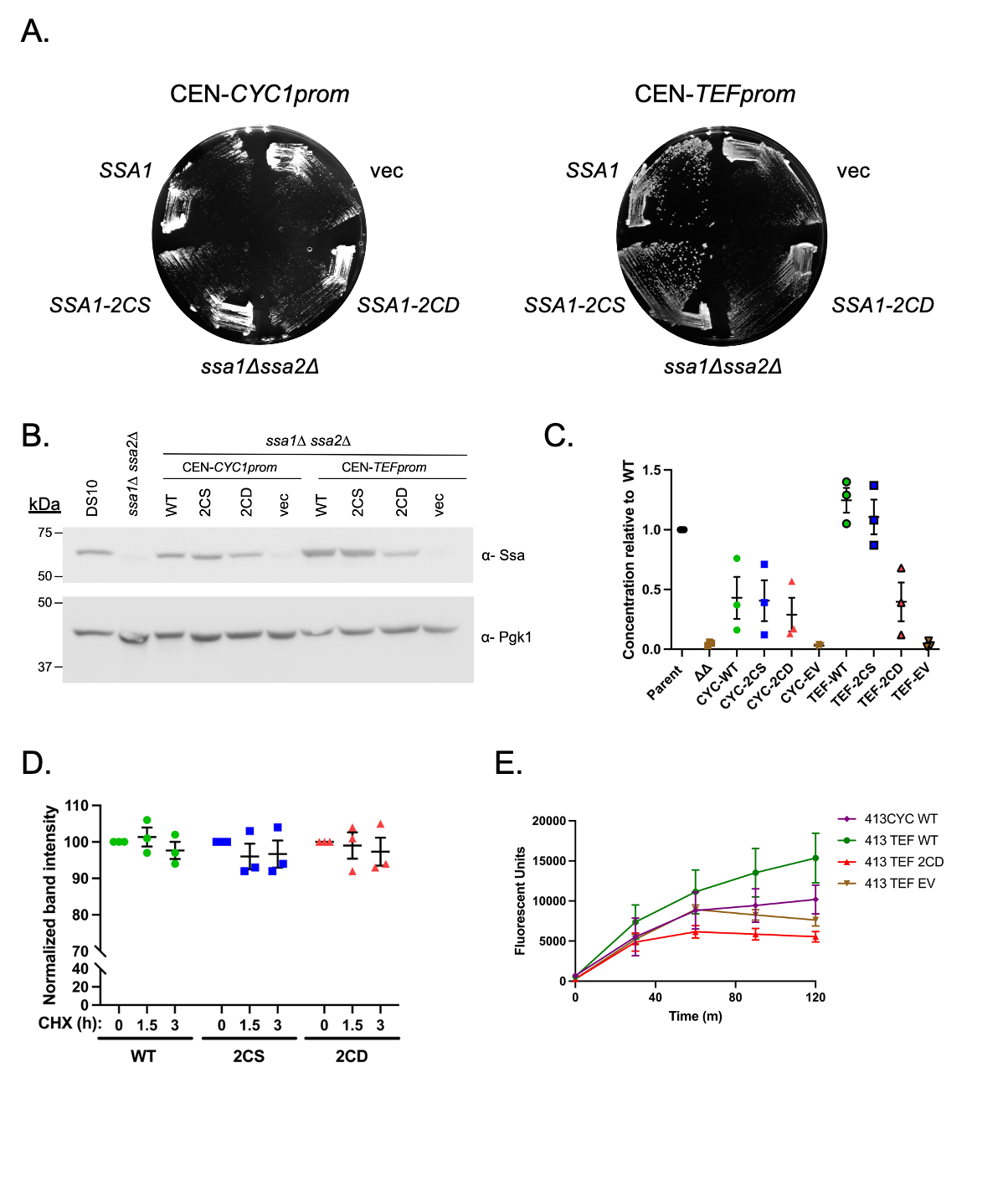
**

**Fig. S3.** **Expression of the Ssa1-2CD protein is restricted relative to wild type Ssa1 and Ssa1-2CS**. (A) 48-hour plate growth of each indicated *SSA* gene driven from the weak *CYC1* promoter and the stronger *TEF* promoter in the *ssa1Δ ssa2Δ* background. (B) Relative protein expression of each indicated Ssa1 protein from the wild type strain DS10, or the respective *CYC1* or *TEF* plasmid in the *ssa1Δ ssa2Δ* background. (C) Quantification of the relative levels of each indicated protein compared to expression in the DS10 background. (D) Immunoblot cycloheximide chase analysis to monitor protein stability over time of each indicated protein driven from the *TEF* promoter, in the *ssa1Δ ssa2Δ* background. (E) *de novo* expression of FFL-GFP. Bolded horizontal bars indicate mean, and error bars indicate SEM.


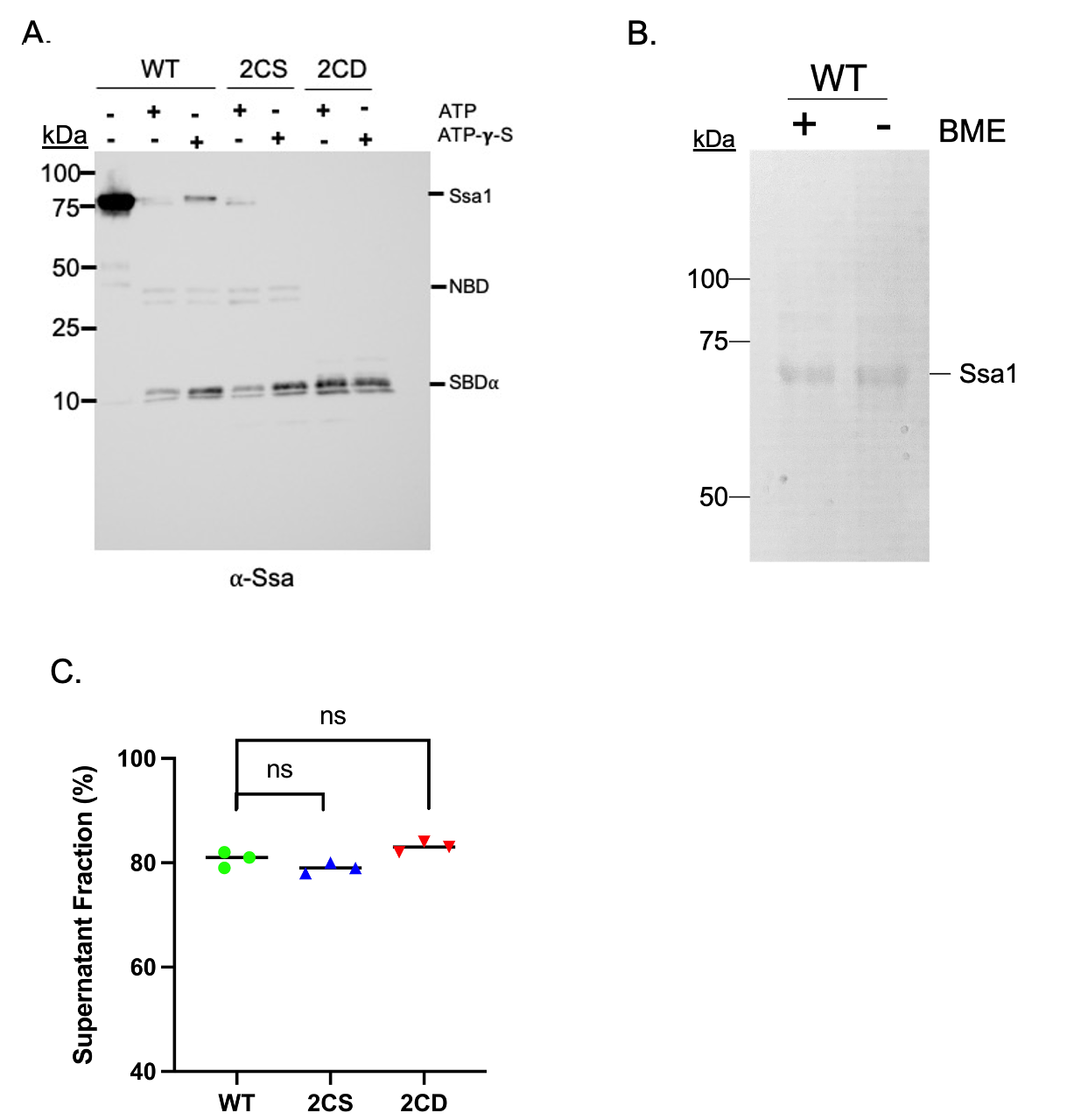


**Fig. S4. Oxidation affects conformation dynamics, but not dimer formation or solubility.**

(A) Limited trypsin fragmentation profile of each indicated Ssa1 incubated with the respective nucleotide. (B) Coomassie staining of Ssa1 WT after exposure to exogenous oxidants as described in *Materials and Methods*, incubated both with and without the reductant beta-mercaptoethanol (BME). (C) Differential centrifugation of the respective Ssa1 protein, reported as percentage of total Ssa1 in the supernatant fraction.


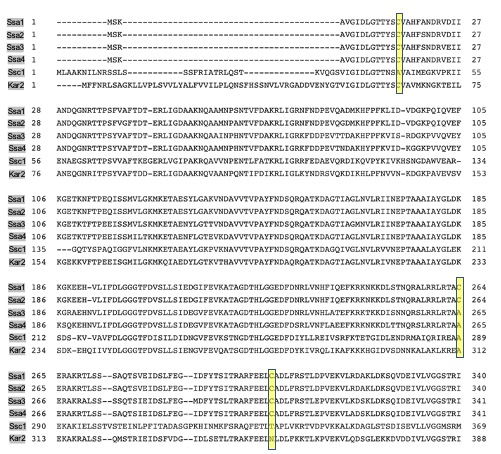


**Fig. S5.** **Reactive cysteines in Hsp70s are not conserved throughout cellular compartments in *S. cerevisiae***

(A) Amino acid alignment of selected Ssa1 homologues found throughout *S. cerevisiae.* The relevant Ssa1 cysteines in this study (C264, C303) and the homologous reside position in other selected Hsp70s are highlighted in yellow.
